# Maternal Caffeine Consumption During Pregnancy and Offspring Cord Blood DNA Methylation: An Epigenome-Wide Association Study Meta-Analysis

**DOI:** 10.2217/epi-2023-0263

**Published:** 2024-02-20

**Authors:** Laura Schellhas, Giulietta S Monasso, Janine F Felix, Vincent WV Jaddoe, Peiyuan Huang, Sílvia Fernández-Barrés, Martine Vrijheid, Giancarlo Pesce, Isabella Annesi-Maesano, Christian M Page, Anne-Lise Brantsæter, Mona Bekkhus, Siri E Håberg, Stephanie J London, Marcus R Munafò, Luisa Zuccolo, Gemma C Sharp

**Affiliations:** 1 School of Psychological Science, University of Bristol, Bristol, BS8 1QU, UK; 2 MRC Integrative Epidemiology Unit at the University of Bristol, Bristol, BS8 2BN, UK; 3 Institute for Sex Research and Forensic Psychiatry, University Medical Center Hamburg-Eppendorf, Hamburg, 20251, Germany[; 4 The Generation R Study Group, Erasmus MC, University Medical Center Rotterdam, Rotterdam, 3015 GD, The Netherlands; 5 Department of Pediatrics, Erasmus MC, University Medical Center Rotterdam, Rotterdam, 3015 GD, The Netherlands; 6 Barcelona Institute for Global Health (ISGlobal), Barcelona, 08003, Spain; 7 Agència de Salut Pública de Barcelona, Pl. Lesseps 1, 08023, Barcelona, Spain; 8 Universitat Pompeu Fabra, Barcelona, 08002, Spain; 9 CIBER Epidemiología y Salud Pública, Madrid, 28029, Spain; 10 INSERM UMR-S 1136, Team of Epidemiology of Allergic and Respiratory Diseases (EPAR), Institute Pierre Louis of Epidemiology and Public Health (IPLESP), Sorbonne University, Paris, 75005, France; 11 Institute Desbrest of Epidemiology and Public Health, INSERM and Montpellier University, Montpellier, 34090, France; 12 Department of Allergic and Respiratory Diseases, Montpellier University Hospital, Montpellier, 34295, France; 13 Department of Physical Health and Aging, Division for Mental and Physical Health, Norwegian Institute of Public Health, Oslo, 0456, Norway; 14 Department of Food Safety, Division of Climate and Environmental Health, Norwegian Institute of Public Health, Oslo, 0456, Norway; 15 PROMENTA Research Centre, Department of Psychology, University of Oslo, Oslo, 0373, Norway; 16 Centre for Fertility and Health, Norwegian Institute of Public Health, Oslo, 0456, Norway; 17 Epidemiology Branch, National Institute of Environmental Health Sciences, NIH, Research Triangle Park, NC27709, USA; 18 NIHR Biomedical Research Centre at the University Hospitals Bristol NHS Foundation Trust and the University of Bristol, Bristol, BS2 8DX, UK; 19 Department of Population Health Sciences, Bristol Medical School, University of Bristol, Bristol, BS8 2PN, UK; 20 Health Data Science Centre, Human Technopole, Milan, 20157, Italy; 21 School of Psychology, University of Exeter, Exeter, EX4 4PY, UK

**Keywords:** caffeine, DNA methylation, epigenetics, offspring health, PACE consortium, pregnancy

## Abstract

**Background:** Prenatal caffeine exposure may influence offspring health via DNA methylation, but no large studies have tested this. **Materials & methods:** Epigenome-wide association studies and differentially methylated regions in cord blood (450k or EPIC Illumina arrays) were meta-analyzed across six European cohorts (n = 3725). Differential methylation related to self-reported caffeine intake (mg/day) from coffee, tea and cola was compared with assess whether caffeine is driving effects. **Results:** One CpG site (cg19370043, *PRRX1*) was associated with caffeine and another (cg14591243, *STAG1*) with cola intake. A total of 12–22 differentially methylated regions were detected with limited overlap across caffeinated beverages. **Conclusion:** We found little evidence to support an intrauterine effect of caffeine on offspring DNA methylation. Statistical power limitations may have impacted our findings.

## Plain language summary

Current guidelines recommend pregnant women to limit caffeine intake to less than 200 mg daily, even though there is no clear proof of its effects on human development. A biological explanation for how exposure to caffeine during pregnancy influences development would help clarify if recommended limits are justified. An epigenetic mechanism, called DNA methylation (DNAm), has been suggested as a potential biological explanation for how caffeine intake during pregnancy influences health development. DNAm can switch genes ‘on’ or ‘off’ in response to environmental influences and therefore act as a bridge between genes and the environment. Studies have found that smoking during pregnancy is connected to over 6000 changes in DNAm at birth, with lasting effects into adulthood. To explore the link between caffeine intake during pregnancy and DNAm at birth, we analyzed data from 3725 mother–child pairs living in different European countries. We looked at effects from coffee, tea and cola intake during pregnancy on children’s DNAm at birth. We found one change in DNAm to be connected to total caffeine and another to cola consumption during pregnancy. These few connections do not provide convincing evidence that caffeine intake during pregnancy impacts children’s DNAm at birth. However, because mothers in our study consumed little caffeine, it is possible that results would be different in studies with participants consuming high amounts of caffeine during pregnancy. Potentially, our study did not include enough people to find very small changes in DNAm that are connected to caffeine consumption during pregnancy.

## Tweetable abstract

EWAS meta-analysis of six European cohorts finds no support for an intrauterine effect of caffeine on DNA methylation at birth. Associations are likely driven by diverse confounding structures of caffeinated drinks, not caffeine itself.

There is growing public and research interest about the effect of caffeine consumption during pregnancy on offspring health. Throughout pregnancy, the metabolic rate of caffeine gradually decreases and in the second and third trimester the half-life of caffeine can be up to four-times longer than outside of pregnancy [1]. Due to pregnancy-related changes to the caffeine metabolism and the potential of caffeine readily crossing the placenta barrier, the European Food Safety Authority states *“…unborn children to be the most vulnerable group for adverse effects of caffeine among the general population”* [2]. Current pregnancy guidelines for caffeine consumption across Asia, Oceania, Europe, Latin America and North America recommend avoiding or limiting caffeine intake during pregnancy [3]. These guidelines are based on evidence from observational study designs that have found associations with an increased risk for low birth weight [4–8], small for gestational age [4,9] and childhood overweight [10]. However, observational studies are likely affected by selection, measurement and confounding biases [11]. Caffeine consumption is a complex phenotype that is culturally bound, with different caffeinated drinks showing varying confounding structures, even across countries that are perceived to be culturally similar (e.g., UK and The Netherlands) [12].

DNA methylation (DNAm) is an epigenetic mechanism that has been proposed to link prenatal caffeine exposure to later health outcomes in offspring [13]. In this epigenetic modification, a methyl group is added to a CpG site in the genome [14]. DNAm undergoes profound changes during embryonic development, making it a valuable proxy for assessing the quality of the intrauterine environment [15]. Animal studies suggest that prenatal caffeine-induced DNAm may have an effect on offspring growth restriction [16], metabolic, cardiac [17,18], neuroendocrine and hormonal [19–21] development. To our knowledge, only one published study today has investigated prenatal caffeine exposure and offspring DNAm in human cord blood. This small study (n = 378) found only one CpG (cg09460369, nearest gene *RAB2A*) to be differentially methylated in association with prenatal exposure to the caffeine metabolite theobromine [22]. A recent epigenome-wide association study (EWAS) meta-analysis of adult populations found coffee consumption to be associated with own peripheral blood DNAm at 11 CpG sites [23]. Discovering associations between maternal caffeine intake during pregnancy and offspring DNAm could provide initial indication for whether DNAm may pose as a potential biological mechanism explaining the associations found in observational studies.

In this international meta-analysis using data from the Pregnancy and Childhood Epigenetics consortium [15], we explored the association between offspring cord blood DNAm and maternal caffeine consumption during pregnancy across different European cultures (UK, The Netherlands, Spain, France and Norway) using different sources of caffeine (from tea, coffee and cola).

## Materials & methods

### Meta-analysis of EWAS

#### Participating cohorts

The EWAS meta-analysis included six independent prospective pregnancy and birth cohorts from the Pregnancy and Childhood Epigenetics consortium [15] that had data available on cord blood DNAm and maternal caffeine consumption during pregnancy. The total sample (n = 3731) included two UK-based cohorts (ALSPAC [24,25] and BiB [26,27]), one Dutch (Generation R) [28,29], one Norwegian (MoBa) [30], one Spanish (INMA) [31] and one French (EDEN) [32] cohort. Recruitment periods varied by cohort and took place between the beginning of the 1990s (ALSPAC) and 2010 (BiB). Ethical approval was obtained by local ethics committees and informed consent for the use of data was obtained from all participants. More details about the individual cohorts can be found in the Supplementary Information.

#### Measurement of maternal caffeine intake during pregnancy

Assessment of maternal caffeine consumption varied by cohort and is described in more detail in Supplementary Information. Generally, mothers self-reported the number of cups they consumed of caffeinated coffee, tea and cola in questionnaires between week 12 and 22 of pregnancy. All cohorts used Food Frequency Questionnaires [33] except for Generation R, which also did not have information on caffeinated cola consumption available. Cups per day were transformed to milligrams of caffeine per day (mg/day), based on the assumption that one standard-sized cup of coffee contains 57 mg, one cup of tea contains 27 mg, and one cup of cola contains 20 mg of caffeine [34]. A continuous total caffeine score was calculated by summing the caffeine content from each caffeinated drink in mg/day (Supplementary Information). Current literature claims that even amounts below the 200 mg/day caffeine limit may have an effect on offspring health and recommends to abstain from caffeine intake during pregnancy [35]. Thus, we investigated, in addition to the continuous score, whether any caffeine exposure (regardless of the amount of caffeine) during pregnancy might have an effect on offspring DNAm. For this analysis, we dichotomized total caffeine into 0 mg/day = none, and >0 mg/day = any.

#### Measurement of DNAm

Cohorts assessed cord blood DNAm data individually, using their own laboratory methods, quality control and normalisation. DNAm data was sampled using the lllumina Infinium^®^ HumanMethylation450 (486,425 probes), except for BiB, which used the Illumina EPIC BeadChip array (Illumina, CA, USA; 866,553 probes). Probes on SNPs, crosshybridizing probes [36] and probes on the sex chromosomes were excluded. In the final meta-analysis, only probes that were available in both arrays (maximum 364,678) were included. Methylation was measured using normalized beta values ranging from 0 to 1, representing 0–100% methylation.

#### Covariates

To adjust for variation in DNAm driven by cell composition, models were adjusted for cell proportions estimated using the Houseman method with a cord blood reference panel [37,38]. Offspring sex was used to conduct sex-stratified sensitivity analyses because sex-specific DNAm differences can still be observed even when restricting analyses to autosomes and removing probes that are crossreactive with sex chromosomes [39]. Another sensitivity analysis was conducted where the unstratified models were additionally adjusted for gestational age at birth. Gestational age could be a mediating factor as it is robustly associated with DNAm [40,41] and there is some evidence that it can be associated with prenatal caffeine exposures [42,43]. To avoid introducing collider bias, we adjusted for gestational age in separate models instead of the main models [44]. To adjust for possible technical variation, all cohorts generated 20 surrogate variables and included them in models – as is standard practice in the field [45].

Each model contained the following covariates [45,46]: an ordinal measure representing maternal education as a proxy for socioeconomic position, maternal age in years, maternal BMI (kg/m^2^), a binary measure of maternal smoking during pregnancy (e.g., in ALSPAC: 0 = no smoking or giving up smoking during the first trimester; 1 = smoking after the first trimester) and a binary assessment of parity (1 = one or more previous children; 0 = no previous children). The Supplementary Information describes the classifications of covariates in each cohort.

### Statistical analyses

#### Cohort-specific statistical analyses

##### Probe-level analysis

The analysis plan and R script is available on GitHub (https://github.com/ammegandchips/Prenatal_Caffeine). Cohorts were asked to exclude multiple pregnancies (e.g., twins) and siblings so that each mother was only included once in the dataset. If cohorts included more than one major ethnic group, they were asked to run the EWAS analysis separately for each group. The EWAS R script included the following: a function to remove probes classified as outliers according to the Tukey method of outlier removal (values <25th percentile - 3 × interquartile range and values >75th percentile + 3 × interquartile range) [47], a function to generate surrogate variables using the R package SVA [48] and a function to run an EWAS of each model using the R package Limma [49]. In a second sensitivity analysis, the binary (any vs none) and total continuous unstratified models were additionally adjusted for gestational age at birth. In a separate sensitivity analysis without gestational age adjustment, the binary (any vs none) and total continuous caffeine models were stratified by offspring’s sex. For quality assurance, an independent shadow meta-analysis was conducted by a coauthor of the University of Bristol.

Prior to meta-analyzing summary results from each cohort, quality checks were conducted to ensure that the EWASs were properly conducted and there were no problems with the data, in line with standard practice in the field [45,50] (Supplementary Information).

##### Differentially methylated regions

The probe level approach was complemented using a regional analysis, which considers DNAm at clusters of neighboring CpG sites throughout the epigenome. This approach is more statistically powerful and arguably more biologically plausible; neighboring CpG sites are assumed to exert similar biological functions. We used the dmrff method [51] to identify differentially methylated regions (DMRs). In this analysis, first candidate DMRs are identified based on the meta-analyzed summary statistic of each CpG site. Candidate DMRs were defined as regions with a minimum of two CpG sites within 500-base pair proximity, which show the same direction of effect and a p-value < 0.05. Second, summary statistics for these candidate DMRs are calculated within each cohort. Last, a meta-analysis is performed based on the cohort DMR summary statistics. The dmrff meta-analysis applies an inverse-variance weighted fixed effects approach that accounts for dependencies between CpG sites [51,52]. Cohorts were supplied with an R script to conduct the DMR analysis using their own data (https://github.com/ammegandchips/Prenatal_Caffeine/blob/master/dmrff.mat.caff.EWAS.cohorts.r). Probes were annotated to the human reference genome version 19, build 37h using the annotation data available from the R package *meffil* [53].

#### Meta-analysis

##### Probe level meta-analysis

Results were meta-analyzed with fixed effect estimates weighted by the inverse of the variance using the software METAL [54]. Multiple testing was accounted for using a 5% false discovery rate [55]. Meta-analyzed results were scrutinized in a similar manner as the individual cohort results and leave-one-cohort-out analysis using the R package *metafor* [56] was performed on the CpGs that showed evidence to be associated with maternal caffeine consumption. Results were deemed to be driven by a single cohort (and therefore to ‘fail’ the leave-one-out test), if the meta-analysis effect estimate changed direction, moved toward the null by more than 20% or had a CI that included 0 after removal of a single cohort.

##### DMR meta-analysis

DMR cohort results were meta-analyzed using an inverse-variance weighted fixed effects approach using the *dmrff.meta* function in the dmrff R package [51]. We defined a DMR as a region with at least two CpG sites with the same direction of effect and a Bonferroni adjusted p-value (P_Bonferroni_) < 0.05.

##### Causal inference & sensitivity analyses

Beverage-specific effects of the meta-analyzed probe-level and DMR results were investigated by comparing the congruence between associations found using different sources of caffeine (that could have different confounding structures). Whereas high congruence between results of the different caffeine models (in terms of CpG site hits and/or genes annotated to CpG sites found in each model) would provide evidence for caffeine being the causal agent driving effects, beverage-specific effects would indicate that factors other than caffeine are driving associations.

To find out which gene pathways are linked to the CpG sites of the caffeine-associated DMRs, a gene ontology (GO) analysis was run using the R package *missMethyl* [57]. We tested enrichment of GO categories and Kyoto Encyclopaedia of Genes and Genomes (KEGG) pathways.

## Results

### Sample characteristics

#### Maternal caffeine consumption during pregnancy

In all cohorts, most mothers (80–94%) consumed at least some caffeine during weeks 1–28 of gestation, with a weighted mean of 85 mg/day over all cohorts, but with large variation within and between cohorts (weighted average standard deviation = 82 mg/day) (Table 1). Approximately 14% of mothers in the total sample consumed more caffeine than the commonly recommended caffeine limit of 200 mg/day. Across all cohorts, coffee and tea were the most common sources of caffeine, with coffee being the most common source in all cohorts except for the UK-based cohorts ALSPAC and BiB, where the most common source was caffeinated tea (Table 1).

**Table 1.** Overview of daily maternal caffeine consumption during pregnancy in the individual cohorts.

Cohort (n)	Weeks of gestation of dietary assessment	Mean total daily caffeine intake (SD)	n users (%) >200 mg of daily caffeine intake^†^	M daily intake of coffee (SD)	M daily intake of tea (SD)	M daily intake of cola (SD)
ALSPAC(n = 729)	18	135 (94)	197 (27)	52 (69)	72 (58)	3 (6)
BiB(Asian; n = 353)	26–28	49 (47)	5 (2)	11 (33)	41 (34)	12 (20)
BiB(White European; n = 306)	26–28	112 (105)	50 (19)	46 (65)	66 (63)	15 (22)
Generation R(n = 798)	18–25	115 (96)	132 (20)	118 (78)	57 (62)	Not available
INMA(n = 378)	12	111 (129)	26 (8)	79 (120)	25 (46)	7 (12)
EDEN(n = 162)	24–28	37 (43)	1 (<1)	25 (42)	6 (15)	6 (9)
MoBa1(n = 999)	22	105 (106)	108 (11)	70 (106)	18 (27)	14 (27)
**Total and % or M and SD**^‡^(n = 3725)	–	85 (82)	519 (14)	46 (65)	26 (35)	5 (11)

† Mothers were grouped as users of caffeine if they indicated to consume more than zero cups of coffee, tea or cola. Caffeine content in milligrams per day.

‡ In the Total row, average caffeine content was calculated by weighting by the inverse variance for each cohort.

M: Mean; SD: Standard deviation.

#### Demographics

A general overview over the demographics of the individual cohorts can be found in Table 2. Except for mothers from BiB, cohorts included slightly more mothers with higher (high school diploma or above) than lower educational attainment. Around 15% of mothers smoked after the second trimester of pregnancy. Mothers who consumed caffeine during pregnancy were about twice as likely to have smoked during pregnancy (20%), compared with mothers who did not consume caffeine during pregnancy (11%) (Supplementary Table 1). Furthermore, mothers who consumed caffeine were more likely to already have children (49%) compared with mothers who did not consume caffeine during pregnancy (33%) (Supplementary Table 1).

**Table 2.** Overview of demographic information across the individual cohorts.

Cohort	Country and ancestry	DNA methylation array	n high maternal socioeconomic position^†^ (%)	M maternal age (SD)	n maternal smoking (%)^‡^	n parity > 0 (%)	M BMI	M gestational age (weeks)
ALSPAC (n = 729)	UK; northern European	450k	375 (51)	29.79 (4.39)	77 (11)	381 (52)	22.79 (3.63)	39.53 (1.52)
BiB(Asian; n = 353)	UK; Pakistani	EPIC	146 (41)	28.21 (5.37)	9 (3)	249 (71)	25.75 (5.23)	39.17 (1.52)
BiB(White European; n = 306)	UK; northern European	EPIC	125 (41)	26.98 (6.15)	93 (30)	159 (52)	27.10 (6.48)	39.29 (1.88)
Generation R (n = 798)	The Netherlands; northern European	450k	458 (57)	30.15 (4.95)	109 (14)	95 (59)	23.06 (3.64)	40.20 (1.48)
INMA (n = 378)	Spain; southern European	450k	277 (73)	31.55 (4.07)	53 (14)	161 (43)	23.79 (4.44)	41.06 (1.34)
EDEN (n = 162)	France; southern and northern European	450k	113 (70)	31.94 (4.10)	26 (16)	324 (41)	23.52 (4.64)	39.51 (1.33)
MoBa1 (n = 999)	Norway; northern European	450k	761 (76)	29.93 (4.35)	287 (29)	580 (58)	24.02 (4.18)	39.95 (1.56)
**Total or M** (n = 3725)	–	–	2255 (61)	30.01(4.60)	654 (18)	1949 (52)	23.61 (4.12)	39.94 (1.51)

In the Total row, average caffeine content was calculated by weighting by the inverse variance for each cohort.

† High maternal socioeconomic position: maternal education ≥ high school diploma.

‡ Continued smoking during pregnancy. Parity = one or more previous pregnancies.

M: Mean; SD: Standard deviation.

### Association between maternal caffeine consumption & offspring cord blood DNAm

Results of the quality control checks for the individual cohort and meta-analyzed results can be found in Supplementary Figures 1–18. The overall EWAS meta-analysis results of the caffeine models can be found in Table 3. After adjusting for multiple testing, one CpG site (cg19370043, nearest gene *PRRX1*) was negatively associated with total maternal caffeine consumption (estimate = -2.18 × 10^-05^; 95% CI: -2.98 × 10^-05^ to -1.37 × 10^-05^; p = 1.32 × 10^-07^) and one CpG site (cg14591243, nearest gene *STAG1*) with caffeine consumed from cola in mg/day (estimate = 2.78 × 10^-05^; 95% CI: 2.779 × 10^-05^ to 2.781 × 10^-05^; p = 5.59 × 10^-09^) (Table 3). For the caffeinated cola results, drinking one extra cup of cola per day would be associated with a 0.06% increase in DNAm at cg14591243. Only the total maternal caffeine-associated CpG site survived the leave-one-out analysis. For the cola-associated CpG site, the leave-one-out analysis indicated that MoBa was driving the effect (Supplementary Figures 16 & 17).

**Table 3.** A summary of results of each epigenome-wide association study model from the probe-level analysis.

Model^†^	CpGs with false discovery rate-corrected p-value < 0.05	CpGs surviving leave-one-out analysis	Meta-analysis sample size	Genomic inflation factor (λ)^‡^
**Any vs no caffeine**				
All offspring (minimally adjusted)^†^	0	NA	3731	0.97
All offspring (adjusted for covariates)	0	NA	3731	0.97
Female offspring (adjusted for covariates)	0	NA	1797	0.99
Male offspring (adjusted for covariates)	0	NA	1934	1.00
All offspring (adjusted for covariates and gestational age)	0		3731	0.97
**Caffeine in mg/day**				
All offspring (minimally adjusted)^†^	33	NA	3731	1.03
All offspring (adjusted for covariates)	1	1 (100%)	3731	1.00
Female offspring (adjusted for covariates)	0	NA	1797	1.00
Male offspring (adjusted for covariates)	0	NA	1934	1.04
All offspring (adjusted for covariates and gestational age)	0		3731	0.99
**Caffeine from coffee**				
All offspring (adjusted for covariates)	0	NA	2779	1.02
**Caffeine from tea**				
All offspring (adjusted for covariates)	0	NA	3477	1.00
**Caffeine from cola**				
All offspring (adjusted for covariates)	1	0	2610	1.00

† Only adjusted for estimated cell counts and 20 surrogate variables. Covariates: maternal age, maternal smoking, maternal parity, maternal education, maternal BMI, estimated cell counts and 20 surrogate variables.

‡ The genomic inflation factor (λ) estimates the extent of bulk inflation of epigenome-wide association study p-values and the excess false positive rate. 1 = no inflation; >1 some evidence of inflation.

NA: Not applicable.

There is a slight deviation in sample sizes between the results and descriptive information (n = 6 participants) because of removal of data in MoBa between 2018 and 2020 due to withdrawal of consent and/or non-Nordic/non-European ancestry.

#### Beverage-specific effects

**Figure 1.**
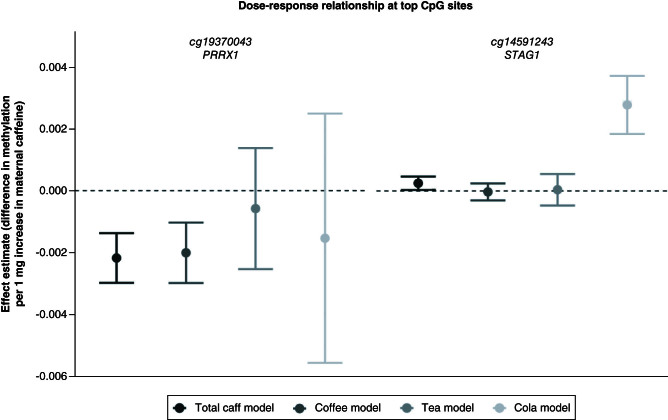
Effect size estimates at the top CpG sites found in the probe-level analysis. Total caff model = total caffeine; Coffee model = caffeine from coffee; Tea model = caffeine from tea; Cola model = caffeine from cola. Error bars represent 95% CIs.

Figure 1 displays DNAm at the two CpG sites discovered in the probe-level analysis across the different caffeine models. If these associations were truly driven by caffeine exposure, we would expect to see similar associations of these CpG sites across the different beverage models. The association between DNAm at cg19370043 and maternal total caffeine consumption appears to be mostly driven by coffee and not by caffeine from tea or cola (Figure 1). For coffee intake specifically, the p-value was < 0.05, but the association with coffee did not survive adjustment for multiple testing in the EWAS meta-analysis, probably because of the lower statistical power to detect small effects in the coffee compared with total caffeine analysis (n total caffeine = 3731 vs n coffee = 2779; because the total caffeine model included mothers with missing beverage-specific data). The association between DNAm at cg14591243 appears to be specific to cola consumption rather than general caffeine consumption.

### DMR meta-analysis

The regional meta-analysis implemented using dmrff detected 22 DMRs for total maternal caffeine consumption with at least two and a maximum of 15 consecutive CpG sites (P_Bonferroni_ < 0.05) (Supplementary Table 2 & Supplementary Figure 19). The strongest evidence was found at a region on chromosome 17, with seven consecutive CpG sites (chr17: 58499679-58499911; estimate = -3.77 × 10^-05^; standard error: 5.02 × 10^-06^; P_Bonferroni_ = 1.42 × 10^-10^; nearest gene *C17orf64*) (Supplementary Table 2). In the any versus no maternal caffeine consumption model, there was evidence for 11 DMRs (Supplementary Table 3). The strongest evidence was found at a region on chromosome 6, with ten consecutive CpG sites (Chr6:31734147-31734554; estimate = -9.44^-03^; standard error: 1.37^-03^; P_Bonferroni_ = 1.93^-06^; nearest gene *C6orf27*). DMR analyses from the individual sources of caffeine revealed 12 DMRs for caffeine consumed from coffee (Supplementary Table 4), 18 DMRs for caffeine from tea (Supplementary Table 5) and 14 DMRs for caffeine consumption from cola (Supplementary Table 6) during pregnancy. The analyses from the sex-stratified models showed evidence for total maternal caffeine being associated with 12 DMRs in cord blood in female sex offspring (Supplementary Table 7) and 18 DMRs in male sex offspring (Supplementary Table 8).

For each pairwise combination of models, we calculated the percentage overlap of CpG sites (or closest gene) by dividing the overlap of CpG sites (or genes) between two models by the sum of the models’ unique CpG sites/genes (e.g., percentage crossover any caffeine and total caffeine models: 7/[167 + 63 - 7] = 0.03 × 100 = 3%). There was very little overlap in CpG sites (range percentage overlap: 0–12%) or annotated genes (percentage overlapping genes: 0–11%) between the different models (Supplementary Table 9).

#### Functional analysis of DMRs

A GO analysis was conducted using the R package *missMethyl* [57]. We tested enrichment of GO categories and KEGG pathways. Neither the functional categories defined by GO terms nor any of the KEGG pathways showed evidence for enrichment in genes annotated to CpG sites in the caffeine-associated DMRs (all false discovery rate adjusted p-values > 0.05). Due to the limited number of DMRs available for the functional analysis, insufficient statistical power may have compromised the identification of meaningful enrichment. The top five KEGG pathways and GO terms with the strongest evidence according to the smallest p-values for each list of CpGs in the caffeine-DMRs are available in Supplementary Table 10.

## Discussion

### Summary & interpretation of findings

We investigated the association of self-reported maternal caffeine consumption during pregnancy with offspring cord blood DNAm using data from six international birth cohorts. For the EWAS meta-analysis, probe-level and regional DMR analyses were applied as hypothesis-free approaches to detect associations between maternal caffeine phenotypes and differential methylation levels in cord blood. We compared caffeine consumption during pregnancy across countries and caffeinated drinks, which reduced the potential for cultural confounding, and analyzed the CpG sites of the maternal caffeine-associated DMRs for their biological function. Results of these analyses show little converging evidence between the associations of the different sources of caffeine, indicating that the associations that we observed are most likely explained by other factors than caffeine exposure.

The probe-level analysis indicated that differences in DNAm at two CpG sites were associated with maternal caffeine intake (one with maternal total caffeine intake, and one with caffeine intake from cola); both showed small effect estimates. According to the genecards database [58], the gene *PRRX1*, which is annotated to the total caffeine-associated CpG site cg19370043, has been found to be associated with determining mesodermal muscle types by regulating muscle creatine kinase. The gene *STAG1*, which has been annotated to the cola-associated CpG site cg14591243, has been found to be associated with sister chromatid cohesion during cell division and diseases, including intellectual developmental disorder and Cornelia de Lange syndrome [58]. The coefficients from the regression analyses represent the change in offspring cord blood DNAm at a given CpG site per 1 mg/day increase in maternal caffeine consumption. Putting these results into real-life context, and assuming causality and linearity of effects, if the recommended limit of caffeine consumption during pregnancy were doubled from 200 mg/day to 400 mg/day, this would only be associated with 0.4% reduction in DNAm at cg19370043. This effect size is in line with the small effect sizes found in the EWAS meta-analysis of adult personal caffeine consumption on DNAm by Karabegović and colleagues [23], where an additional cup of coffee (= 57 mg) was associated with a 0.2% decrease in peripheral blood DNAm at a CpG site near *AHRR*, which would be equivalent to a 0.7% decrease in DNAm per 200 mg/day of caffeine (0.2%/57 mg of caffeine per cup of coffee × 200). These estimated effect sizes appear to be much smaller than the estimated effect of smoking, which is the lifestyle exposure with the strongest effect on DNAm discovered to date. Sustained smoking during pregnancy was associated with changes of up to ∼7% decrease in offspring cord blood DNAm at the *AHRR* gene [46]. As acknowledged by Karabegović *et al.*, because their smoking adjustment did not include the amount of smoking or duration of smoking, the coffee-associated DNAm differences might be explained by residual confounding by smoking [23].

In the regional analyses, we identified 12–22 DMRs for each of the caffeine models. Yet, lack of congruence of associations across models, which was evident in the probe-level and regional analysis, provided evidence for beverage-specific effects instead of the effects being driven by caffeine (which is common to all included beverages).

### Strengths & limitations

This was the first large international EWAS meta-analysis investigating associations between offspring DNAm and caffeine from coffee, tea and cola during pregnancy. A major strength of this study is the consideration of the effects of other common sources of caffeine besides coffee. Consumption of the different sources of caffeine might be differentially socially patterned, allowing capturing a larger spectrum of the caffeine-consuming population. For instance, British and non-European ethnicities consume more caffeinated tea than coffee [12,59]. Further, it is indicated that the main source of caffeine might change during pregnancy, with even habitual coffee drinkers preferring caffeinated tea to coffee during pregnancy [60,61]. Last, in contrast to previous research, this study assessed maternal caffeine consumption through mg/day instead of cups per day, which is a useful approach to isolate the effect of caffeine, and allow comparison between different caffeinated beverages and a more fine-tuned assessment of the effects of different caffeine dosages. We also adjusted for maternal smoking, an important potential confounder in analyses of caffeine intake, along with other potential confounding variables.

The findings should be considered in the light of the following limitations. Caffeine assessment in the meta-analysis relied on self-report, which might be underestimated [62] or underreported during pregnancy because of social stigma around maternal health behaviors [63,64]. However, this would be more obvious for more recent cohorts and less and less likely for older cohorts, such as ALSPAC, due to awareness of the potential toxicity of caffeine in pregnancy emerging only recently. Most of the cohorts of this study were assessed in the beginning of 2000s except for ALSPAC, which was assessed in the beginning of the 1990s. Caffeine consumption across the included cohorts does not show a clear pattern of change over time. There is a lack of nationally representative studies for caffeine consumption during pregnancy but a systematic review of caffeine consumption in the general population found that consumption remained stable between 1997 and 2015 [65]. The questions used to assess caffeine consumption in the cohorts only allowed for rough estimations of maternal caffeine consumption [66], which could reduce power. Generalizability of the results to other populations might be limited by examining only second trimester consumption, the relatively low caffeine intake and the tendency for birth cohorts to enroll more advantaged families [25]. Effects of caffeine exposure during pregnancy on offspring cord blood DNAm were only assessed at regions available on the 450k array, which only covers around 2% of CpG sites of the entire epigenome [67]. Thus, differentially methylated CpGs or regions not covered by the array may have been missed in this study. We only assessed offspring DNAm in blood. There is some evidence suggesting that DNAm levels in blood might be able to proxy for DNAm levels in other tissues, yet we cannot rule out that maternal caffeine during pregnancy might be influencing DNAm differentially in other tissue types [68]. Finally, this study indicates that, if maternal caffeine consumption influences cord blood DNAm, the effect is likely to be small. Although our meta-analysis maximized sample sizes, even larger sample sizes with more variable levels of caffeine consumption may be required to detect small effects of prenatal caffeine exposure on offspring DNAm. Due to the dynamic nature of DNAm, results of EWAS are prone to capture associations of confounding variables [69]. Though attempts were made to reduce confounding, we cannot rule out that confounding influenced the results.

### Future research

Future research should aim to use a more accurate assessment of caffeine consumption during pregnancy by considering differing types of coffee, brewing times and cups sizes, and/or assessing biomarkers of caffeine such as plasma concentrations of the caffeine metabolite paraxanthine [70]. Also, future research should investigate the effects of high caffeine consumption on offspring DNAm (e.g., comparison of above vs below the commonly recommended limit of 200 mg/caffeine). Triangulation strategies may be applied to disentangle confounded from causal effects of caffeine exposure during pregnancy on offspring DNAm. These might include further exploring the different confounding structures of various caffeinated beverages and considering individual differences in the maternal metabolism of caffeine. Maternal caffeine metabolism might influence intensity of exposure during pregnancy and might change the effect of caffeine on offspring DNAm. For instance, studies could conduct analyses using genetic variants that account for differences in caffeine metabolism [71] and/or consider prepregnancy caffeine consumption to account for differences in the tolerance to effects of caffeine during pregnancy [66]. Also, more assessments of prenatal paternal caffeine consumption would enable the conduction of negative control analyses to investigate intrauterine effects [72,73], as well as investigating the effects of paternal caffeine consumption prior to pregnancy and its effect on offspring DNAm in its own right [73].

## Conclusion

In conclusion, results of this large scale EWAS meta-analysis indicate little evidence for a strong association between maternal caffeine consumption during the second trimester of pregnancy and offspring cord blood DNAm.

**Summary points**

- This large-scale meta-analysis of epigenome-wide association studies across six European cohorts does not support an intrauterine effect of caffeine on offspring cord blood DNA methylation.
- Lack of overlap between associations with different caffeinated drinks suggest that any (weak) associations were driven by diverse confounding structures of different caffeinated drinks, rather than caffeine *per se*.
- More research is needed to understand the biological mechanisms driving potential effects of caffeine on offspring health.

## Supplementary data

To view the supplementary data that accompany this paper please visit the journal website at: www.tandfonline.com/doi/suppl/10.2217/epi-2023-0263

Supplemental material This material has been provided by the authors to give readers additional information about their work.
